# Effect of glycemic control on lymphocyte subsets in the dissemination of pulmonary tuberculosis: A retrospective analysis

**DOI:** 10.1016/j.imj.2025.100183

**Published:** 2025-05-17

**Authors:** Yujun Lin, Xiaohong Chen, Jiangwei Chen, Di Wu

**Affiliations:** a Department of Respiratory and Critical Care Medicine, Fuzhou Pulmonary Hospital of Fujian Province, Fuzhou 350008, Fujian Province, China; b Department of Tuberculosis, Fuzhou Pulmonary Hospital of Fujian Province, Fuzhou 350008, Fujian Province, China; c The Medical Services Section, Fuzhou Pulmonary Hospital of Fujian Province, Fuzhou 350008, Fujian Province, China

**Keywords:** Pulmonary tuberculosis, Extrapulmonary tuberculosis, Glycated hemoglobin, Lymphocyte subpopulation, Immunity

## Abstract

**Background:**

Extrapulmonary tuberculosis (EPTB) complicates pulmonary tuberculosis (PTB) management. Diabetes mellitus impairs immune function, worsening tuberculosis (TB) outcomes.

**Methods:**

This retrospective study investigates the effect of glycemic control on immune function and TB dissemination in 1,768 TB patients (2022–2024). Patients were stratified by glycated hemoglobin (HbA1c) levels (≤ 6% vs. > 6%) and fasting blood glucose (FBG) concentrations (< 7 vs. ≥ 7 mmol/L). Lymphocyte subsets (CD3^+^, CD4^+^, CD8^+^ T cells, CD19^+^ B cells, and CD16^+^CD56^+^ natural killer cells) were compared between glycemic control and TB groups. Multiple regression and threshold effect analysis were conducted to assess the effects of HbA1c and CD3^+^ T cells on TB dissemination and their critical values.

**Results:**

Poor glycemic control was associated with lower cell counts of all lymphocyte subsets in patients with PTB (all *p* < 0.0001). Similar reductions were observed in patients with concurrent PTB and EPTB (PTB + EPTB) when HbA1c values > 6% (all *p* < 0.05). When HbA1c values ≤ 6% or FBG concentrations < 7 mmol/L, patients with PTB + EPTB showed lower immune cell counts than PTB (*p* < 0.05). Multiple regression indicated HbA1c increased TB dissemination risk (OR = 10.95), while CD3^+^ T cells showed protective effects. Threshold effect analysis identified an HbA1c values ≥ 7.4% for metabolic control and CD3^+^ T cell thresholds of 387/µL (immune deficiency) and 2,100/µL (immune overactivation).

**Conclusions:**

Poor glycemic control impairs immune cells, while EPTB further reduces immune cell numbers. Integrated glycemic management and immunological monitoring help optimize treatment strategies and improve clinical outcomes, particularly in patients at risk for EPTB.

## Highlights

• Poor glycemic control significantly impairs lymphocyte counts in TB patients.
• EPTB further reduces immune cell populations, even with good glycemic control.
• HbA1c ≥ 7.4% identified as critical threshold for increased TB dissemination risk.
• CD3^+^ *T* cells show protective effects with optimal range between 387 and 2,100/µL.
• Integrated glycemic-immune monitoring optimizes treatment in diabetes-TB comorbidity.

## Graphical abstract

**Figure:**
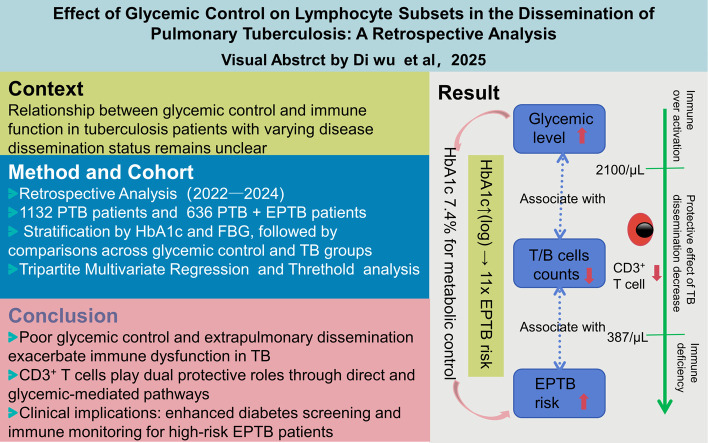

## 1 Introduction

Tuberculosis (TB)^1^ is a severe respiratory infectious disease caused by *Mycobacterium tuberculosis.*^2^ In 2023, TB became the leading cause of death from a single infectious agent^3^. Therefore, there is an urgent need for stronger political commitment to cure TB. TB primarily manifests as pulmonary tuberculosis (PTB), which accounts for > 80% of all cases. However, *Mycobacterium tuberculosis* can disseminate throughout the body, leading to extrapulmonary tuberculosis (EPTB), which involves organs outside the lungs^4^. Currently, the incidence of EPTB among TB cases ranges from 14% to 53%.^5^, ^6^, ^7^, ^8^, ^9^ Patients with concurrent PTB and EPTB (PTB + EPTB) are more likely to have comorbidity^9^ and often require a longer hospital stay and higher treatment costs than patients with PTB alone.^10^^,^^11^ Additionally, management of EPTB is less well addressed than that of PTB by TB control programs in developing countries.^12^ The occurrence, progression, and prognosis of PTB and EPTB are strongly affected by the patient**'**s immune status.^13^

The comorbidity of diabetes in patients with TB has become increasingly prevalent. Co-occurrence of diabetes mellitus (DM) with TB was found in approximately 0.4 million people in 2021 worldwide.^14^ This number is likely to increase because of the rising incidence of diabetes caused by obesity.^15^ Patients with diabetes show altered proliferation of T cells, and B cells reflect dysregulation in immunity.^16^^,^^17^ Additionally, the comorbidity of TB and DM complicates the treatment of TB because TB-induced inflammation and metabolic changes can contribute to insulin resistance and the development of type 2 diabetes, suggesting a bidirectional relationship.^18^ While previous studies have shown a reduction in lymphocyte subsets in patients with TB or DM, whether diabetes further suppresses immunity in patients with TB is unclear, and this situation could promote the dissemination of EPTB from PTB.

Therefore, this study aimed to analyze peripheral blood lymphocyte counts in patients with PTB and in those with PTB + EPTB under different glycemic control conditions (HbA1c values ≤ 6% vs. > 6% and fasting blood glucose [FBG] concentrations < 7 vs. ≥ 7 mmol/L). By examining the differences in immune status across varying levels of glycemic control and TB sites, we hope to enhance the understanding of the associations between diabetes, immune function, and the pathogenesis of PTB complicated by EPTB.

## 2 Materials and methods

### 2.1 Study design and participants

#### 2.1.1 Study subjects and grouping

From 1 January 2022 to 31 June 2024, 1,132 patients with PTB and 636 with PTB + EPTB were diagnosed at Fuzhou Pulmonary Hospital of Fujian Province, China, by comprehensive clinical assessment. The patients were stratified by HbA1c levels (≤ 6% vs. > 6%) and FBG concentrations (< 7 vs. ≥ 7 mmol/L). The PTB + EPTB group was subdivided into three subgroups: PTB with tuberculous pleuritis (PTB + TBP); PTB with bronchial tuberculosis (PTB + BTB), and PTB with miscellaneous extrapulmonary tuberculosis (PTB + M-EPTB), which included extrapulmonary cases excluding TBP and BTB. M-EPTB in this study encompassed tuberculous meningitis, osteoarticular TB, intestinal TB, extrapulmonary lymph node TB, cutaneous TB, tuberculous pericarditis, and multiple serosal cavity TB.

#### 2.1.2 Inclusion criteria

The inclusion criteria were: (1) patients with PTB or PTB + EPTB diagnosed in accordance with the Definitions and Reporting Framework for Tuberculosis—2013 revision (updated 2014 and 2020)^4^; and (2) newly diagnosed patients without prior anti-TB treatment.

#### 2.1.3 Exclusion criteria

The exclusion criteria were as follows: (1) human immunodeficiency virus or viral hepatitis; (2) endocrine/metabolic diseases (excluding DM) such as hyperthyroidism; (3) rheumatic diseases such as systemic lupus; (4) tumors or administration of immunosuppressants; (5) other lung conditions (e.g. infections, chronic obstructive pulmonary disease, and asthma); (6) pregnancy or lactation; (7) non-tuberculous mycobacterial infections; and (8) having more than 1 month of irregular TB treatment, treatment failure, or recurrence.

### 2.2 Methods

#### 2.2.1 Collection of clinical data

Clinical data included sex, age, smoking status, and diagnosis of TB. Examinations included acid-fast staining (AFS), absolute counts of CD3^+^, CD4^+^, and CD8^+^ T cells, CD19^+^B cells, CD16^+^CD56^+^ natural killer (NK) cells, neutrophils, HbA1c levels, FBG concentrations, albumin concentrations, and adenosine deaminase (ADA) concentrations in peripheral blood. The specimens were collected from the time of admission until before initiating anti-TB treatment.

#### 2.2.2 Statistical analysis

Clinical characteristics description was conducted using EasyStat software (X&Y solutions, Inc. Version 5.0, Boston, MA, USA) and R software (The R Foundation for Statistical Computing, version 4.3.2, Vienna, Austria), continuous variables were tested for normality, and natural logarithmic transformation was applied to normalize the distribution of non-normally distributed variables, including HbA1c, fasting blood glucose (FBG), immune cell counts (CD3^+^ T cells, CD4^+^ T cells, CD8^+^ T cells, CD19^+^ B cells, and NK cells), neutrophils, albumin, and ADA. Comparisons of continuous variables across groups were performed using the Kruskal–Wallis rank-sum test. Categorical variables, such as sex, smoking status, and AFS results, were analyzed using Fisher's exact test. Statistical analyses between two groups were performed using GraphPad Prism software (version 9.5, GraphPad Software, San Diego, CA, USA).The Shapiro–Wilk test was used to assess the normality of the data distribution. the independent *t*-test was used for normally distributed data. The Mann–Whitney *U* test was applied for non-normally distributed data. Multiple regression analysis and threshold effect analysis were conducted using EasyStat software and R software to assess the effects of HbA1c and CD3^+^ T cells on the risk of TB dissemination and to determine their critical values. A significance level of *p* < 0.05 was used for determining statistical significance.

## 3 Results

### 3.1 Analysis of clinical characteristics of PTB and EPTB

The clinical characteristics of the patients are shown in Table 1. A total of 1,768 patients with TB, including 1,132 with PTB and 636 who also had various forms of EPTB, were enrolled in this study. There were significant differences in demographic and clinical characteristics between the groups. The mean age ranged from 46.50 ± 8.55 years in the PTB + BTB group to 52.39 ± 17.76 years in the PTB group. Male predominance was observed in most groups except for the PTB + BTB group in which women accounted for 53.30% of cases. The prevalence of smoking varied significantly, with the highest percentage in the PTB + BTB group and the lowest in the PTB group.

**Table 1:** Clinical characteristics of patients with PTB and EPTB.

Characteristics	PTB	PTB + EPTB	PTB + EPTB			*p*
			PTB + TBP (*n* = 361)	PTB + BTB (*n* = 197)	PTB + M-EPTB	
*N*	1132	636	361	197	78	
Age (years)	52.39 ± 17.76	50.08 ± 19.70	52.14 ± 20.61	46.50 ± 18.55	49.54 ± 16.87	< 0.001^a^
Sex, *n* (%)						< 0.001^b^
Female	260 (22.97)	192 (30.19)	63 (17.45)	105 (53.30)	24 (30.77)	
Smoking status, *n* (%)						< 0.001^b^
Yes	626 (55.30)	422 (66.35)	227 (62.88)	152 (77.16)	43 (55.13)	
No	506 (44.70)	214 (33.65)	134 (37.12)	45 (22.84)	35 (44.87)	
HbA1c	−2.80 ± 0.26	−0.65 ± 1.23	−1.57 ± 1.40	0.06 ± 0.02	0.06 ± 0.02	< 0.001^a^
FBG	1.76 ± 0.35	1.72 ± 0.29	1.73 ± 0.27	1.67 ± 0.27	1.80 ± 0.35	< 0.001^a^
CD3^+^T cell	6.94 ± 0.56	6.76 ± 0.54	6.63 ± 0.56	6.97 ± 0.43	6.82 ± 0.50	< 0.001^a^
CD4^+^*T* cell	6.38 ± 0.61	6.20 ± 0.58	6.06 ± 0.61	6.42 ± 0.45	6.27 ± 0.56	< 0.001^a^
CD8^+^*T* cell	5.94 ± 0.63	5.74 ± 0.63	5.63 ± 0.65	5.95 ± 0.55	5.76 ± 0.63	< 0.001^a^
CD19^+^B cell	5.14 ± 0.88	5.01 ± 0.77	4.86 ± 0.81	5.25 ± 0.65	5.11 ± 0.71	< 0.001^a^
NK cell	5.20 ± 0.76	5.10 ± 0.72	5.05 ± 0.72	5.18 ± 0.70	5.14 ± 0.74	0.007^a^
Neutrophil	1.48 ± 0.50	1.73 ± 1.38	1.45 ± 0.45	1.44 ± 0.48	3.75 ± 3.05	< 0.001^a^
Albumin	38.53 ± 5.01	37.36 ± 4.68	36.21 ± 4.51	39.32 ± 3.98	37.51 ± 5.41	< 0.001^a^
ADA	2.56 ± 0.42	4.91 ± 5.70	2.68 ± 0.37	5.03 ± 6.66	14.24 ± 5.96	< 0.001^a^
AFS, *n* (%)						< 0.001^b^
Negative	843 (74.47)	500 (78.62)	328 (90.86)	109 (55.33)	63 (80.77)	
Positive	289 (25.53)	136 (21.38)	33 (9.14)	88 (44.67)	15 (19.23)	

*Notes*: To address the non-normal distribution of laboratory parameters (HbA1c, FBG, CD3^+^ T cells, CD4^+^ T cells, CD8^+^ T cells, CD19^+^ B cells, NK cells, neutrophils, albumin, and ADA), natural logarithmic transformation was applied to normalize the distribution. Data are presented as the mean ± standard deviation or *n* (%). Data were analyzed by the ^a^Kruskal–Wallis rank sum test or ^b^Fisher's exact test.

*Abbreviations*: PTB, pulmonary tuberculosis; EPTB, extrapulmonary tuberculosis; TBP, tuberculous pleuritis; BTB, bronchial tuberculosis; M-EPTB, miscellaneous EPTB; FBG, fasting blood glucose; ADA, adenosine deaminase; AFS, acid-fast staining.

Regarding immunological parameters, T lymphocyte subsets showed distinct patterns across the groups. The PTB + BTB groups showed higher levels of CD3^+^, CD4^+^, and CD8^+^ T cells than the other groups (all *p* < 0.001). B cells (CD19^+^) were highest in the PTB + BTB group and lowest in the PTB + TBP group (*p* < 0.001).

Notable differences were observed in clinical parameters. The PTB + M-EPTB group showed a markedly elevated neutrophil count and ADA concentrations then the other groups. Albumin concentrations were highest in the BTB group and lowest in the PTB + TBP group. The positivity rate of AFS varied significantly, with the PTB + BTB showing the highest positive rate and the PTB + TBP had the lowest (*p* *<* 0.001).

### 3.2 Changes in T, B, and NK cell subset numbers in the PTB and PTB + EPTB groups at different HbA1c levels and FBG concentrations

**Fig. 1:**
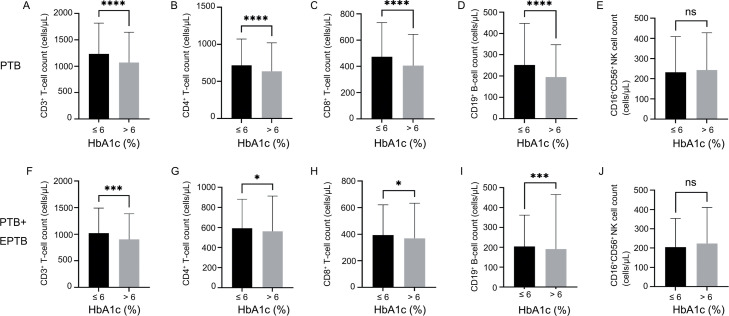
Comparison of TBNK subset cell counts at the time of admission at different HbA1c levels in the PTB and PTB + EPTB groups. *p < 0.05; ^⁎⁎⁎^*p* < 0.001; ^⁎⁎⁎⁎^*p* < 0.0001; ns, not significant. *Abbreviations*: HbA1c, glycated hemoglobin; PTB, pulmonary tuberculosis; EPTB, extrapulmonary tuberculosis.

In patients with PTB, the HbA1c > 6% group showed significantly lower counts of CD3^+^ T cells (964 vs. 1,166, *p* < 0.0001), CD4^+^ T cells (573 vs. 683, *p* < 0.0001), CD8^+^ T cells (356 vs. 440, *p* < 0.0001), and CD19^+^ B cells (116 vs. 211, *p* < 0.0001) than the HbA1c ≤ 6% group (Fig. 1A–D). In contrast, the CD16^+^CD56^+^ NK cell count was not significantly different between these two groups (182 vs. 178, *p* = 0.7171) (Fig. 1E).

A similar pattern of cell counts to that in patients with PTB was observed in patients with PTB + EPTB. The HbA1c > 6% group showed significantly lower counts of CD3^+^ T cells (790 vs. 988, *p* = 0.001), CD4^+^ T cells (463 vs. 550, *p* = 0.0230), CD8^+^ T cells (294 vs. 360, *p* = 0.0117) and CD19^+^ B cells (145 vs. 171, *p* = 0.0006) than the HbA1c ≤ 6% group (Fig. 1F–I). The CD16^+^CD56^+^ NK cell count was not significantly different between these two groups (170 vs. 163, *p* = 0.6867) (Fig. 1J).

We then analyzed the changes in T, B, and NK (TBNK) cell subsets at different HbA1c levels in the PTB with EPTB subgroups. As showed in Supplementary result 1, Analyses of the EPTB subgroups showed a similar pattern of a reduction in immune cells under poor glycemic control (Fig. S1).

To further investigate the effect of glycemic control on TBNK cell subsets in patients with TB, we incorporated FBG as an additional glycemic control parameter. As showed in Supplementary result 2, in patients with PTB, the FBG ≥ 7 mmol/L group showed significantly lower counts of CD3^+^ T cells (940 vs. 1147, *p* < 0.0001), CD4^+^ T cells (541 vs. 675, *p* < 0.0001), CD8^+^ T cells (341 vs. 433, *p* < 0.0001), and CD19^+^ B cells (165 vs. 207, *p* < 0.0001) than the FBG < 7 mmol/L group (Fig. S2A–D). However, the CD16^+^CD56^+^ NK cell count was not significantly different between these two groups (176 vs.184, *p* = 0.3487) (Fig. S2E).

A similar pattern of cell counts to that in patients with PTB was observed in those with PTB + EPTB. The FBG ≥ 7 mmol/L group appeared to show lower counts of CD3^+^ T cells (815 vs. 960, *p* = 0.1047), CD4^+^ T cells (490 vs. 526, *p* = 0.0978), CD8^+^ T cells (306 vs. 334, *p* = 0.1334), and CD19^+^ B cells (156 vs.161, *p* = 0.3943) than the FBG < 7 mmol/L group, but this was not significant. There was also no significant difference in the CD16^+^CD56^+^ NK cell count between these two groups (174 vs. 163, *p* = 0.5346).

### 3.3 Comparison of TBNK cell subsets between the PTB and PTB + EPTB groups with HbA1c levels ≤ 6% and FBG concentrations < 7 mmol/L

**Fig. 2:**
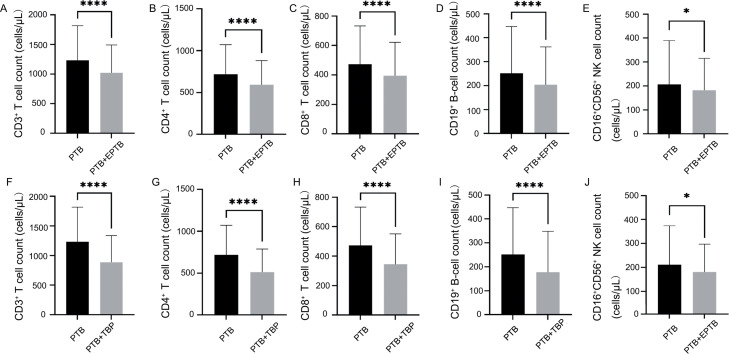
Comparison of TBNK subset cell counts between the PTB and PTB + EPTB groups and between the PTB and PTB + TBP groups when HbA1c values were ≤ 6%. *p < 0.05; ^⁎⁎⁎⁎^*p* < 0.0001. PTB, pulmonary tuberculosis; EPTB, extrapulmonary tuberculosis; TBP, tuberculosis pleuritis.

When HbA1c levels were ≤ 6%, the PTB + EPTB group showed significantly lower counts of CD3^+^ T cells (988 vs. 1166, *p* < 0.0001), CD4^+^ T cells (550 vs. 683, *p* < 0.0001), CD8^+^ T cells (360 vs. 440, *p* < 0.0001), CD19^+^ B cells (171 vs. 211, *p* < 0.0001), and CD16^+^CD56^+^ NK cells (163 vs.182, *p* = 0.0113) than the PTB group (Fig. 2A–E). Furthermore, the PTB + TBP subgroup showed significantly lower counts of CD3^+^ T cells (786 vs. 1,166, *p* < 0.0001), CD4^+^ T cells (467 vs. 683, *p* < 0.0001), CD8^+^ T cells (303 vs. 440, *p* < 0.0001), CD19^+^ B cells (144 vs. 211, *p* < 0.0001), and CD16^+^CD56^+^ NK cells (166 vs. 182, *p* = 0.0113) than the PTB group (Fig. 2F–J).

The effect of glycemic control on TBNK cell subsets in PBT and EBTB was further assessed by incorporating FBG measurements as an additional metabolic parameter. As showed in Supplementary result 3, When FBG concentrations were < 7 mmol/L, the PTB + EPTB group showed significantly lower counts of CD3^+^ T cells (960 vs. 1,147, *p* < 0.0001), CD4^+^ T cells (526 vs. 675, *p* < 0.0001), CD8^+^ T cells (334 vs. 433, *p* < 0.0001), CD19^+^ B cells (161 vs. 207, *p* < 0.0001), and CD16^+^CD56^+^ NK cells (162 vs. 184, *p* = 0.0018) than the PTB group (Fig. S3A–E). Furthermore, the PTB + TBP subgroup showed significantly lower counts of CD3^+^ T cells (774 vs. 1,147, *p* < 0.0001), CD4^+^ T cells (461 vs. 675, *p* < 0.0001), CD8^+^ T cells (290 vs. 433, *p* < 0.0001), CD19^+^ B cells (144 vs. 207, *p* < 0.0001), and CD16^+^CD56^+^ NK cells (161 vs. 184, *p* = 0.0113) than the PTB group (Fig. S3F–J).

Analyses of the other subgroups of EPTB (PTB + BTB and PTB + M-EPTB groups) under good glycemic control are shown in the Supplementary result 4 (Fig. S4).

### 3.4 Comparison of TBNK cell subsets between the PTB and PTB + EPTB groups with HbA1c levels > 6% and FBG concentrations ≥ 7 mmol/L

**Fig. 3:**
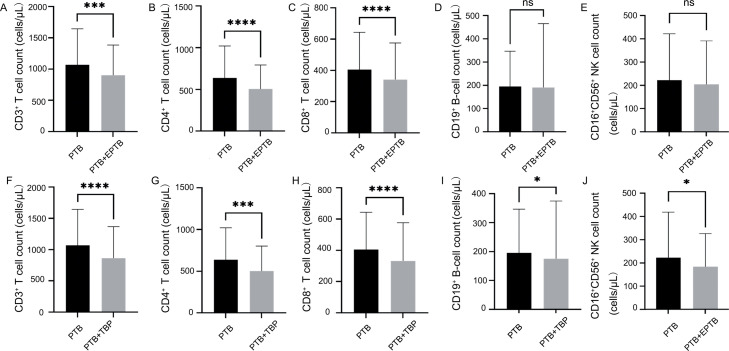
Comparison of TBNK subset cell counts between the PTB and PTB + EPTB groups and between the PTB and PTB + TBP groups when HbA1c levels were > 6%. *p < 0.05; ^⁎⁎⁎^*p* < 0.001; ^⁎⁎⁎⁎^*p* < 0.0001; ns, not significant. *Abbreviations*: PTB, pulmonary tuberculosis; EPTB, extrapulmonary tuberculosis; TBP, tuberculosis pleuritis.

The PTB + EPTB group showed significantly lower counts of CD3^+^ T cells, CD4^+^ T cells, and CD8^+^ T cells than the PTB group when HbA1c levels were > 6% (790 vs. 964, *p* = 0.0005; 444 vs. 573, *p* < 0.0001; 282 vs. 356, *p* < 0.0001, respectively; Fig. 3A–C). However, the counts of CD19^+^ B cells and CD16^+^CD56^+^ NK cells were not significantly different between these two groups (145 vs. 166, *p* = 0.0790; 170 vs. 178, *p* = 0.1281; Fig. 3D and E).

However, the PTB + TBP subgroup showed significantly lower counts of CD3^+^ T cells, CD4^+^ T cells, CD8^+^ T cells, CD19^+^ B cells, and CD16^+^CD56^+^ NK cells than the PTB group when HbA1c levels were > 6% (747 vs. 964, *p* < 0.0001; 447 vs. 573, *p* < 0.0001; 268 vs. 356, *p* < 0.0001; 141 vs. 166, *p* = 0.0190; 160 vs. 178, *p* = 0.0172, respectively; Fig. 3F–H).

To further determine the effects of the glycemic status on TBNK lymphocyte subsets in patients with PTB and EPTB, FBG was used as a complementary glycemic control marker. When FBG concentrations ≥ 7 mmoL/L the PTB + EPTB group appeared to show lower counts of CD3^+^ T cells, CD4^+^ T cells, CD8^+^ T cells, CD19^+^ B cells, and CD16^+^CD56^+^ NK cells (898 vs. 940, *p* = 0.1212; 490 vs. 541, *p* = 0.1014; 306 vs. 341, *p* = 0.1104; 156 vs. 165, *p* = 0.6278; 174 vs. 176, *p* = 0.7614, respectively) than the PTB group, but these results were not significant. However, as showed in Supplementary result 5, the PTB + TBP subgroup showed significantly lower counts of CD3^+^ T cells, CD4^+^ T cells, CD8^+^ T cells, and CD19^+^ B cells than the PTB group when FBG concentrations were ≥ 7 mmol/L (755 vs. 940, *p* = 0.0050; 451 vs. 541, *p* = 0.0057; 262 vs. 341, *p* = 0.0323; 139 vs. 165, *p* = 0.0492, respectively; Fig. S5A–D). The PTB + TBP subgroup also appeared to show a lower count of CD16^+^CD56^+^ NK cells,but this was not significant (172 vs. 176, *p* = 0.0172; Fig. S5E).

Analyses of the other subgroups of EPTB (BTB and M-EPTB) under poor glycemic control are shown in the Supplementary result 6 (Fig. S6).

### 3.5 Bidirectional glycemic–immune regulation in TB dissemination

To determine the associations between glycemic control, immune function, and TB dissemination, we conducted a tripartite regression analysis (Table 2). Model I examined the effect of ln HbA1c on the TB type (PTB vs. PTB + EPTB), Model II assessed the effect of the CD3^+^ T cell count on the TB type, and Model III examined how the CD3^+^ T cell count affected categorized HbA1c levels (cutoff at ln[6%] = −2.813). All continuous variables underwent logarithmic transformation for normality. Adjusted confounding factors are shown in the footnote of Table 2.

**Table 2:** Tripartite regression analysis of glycemic–immune crosstalk in TB dissemination.

Model	Exposure	Outcome	Non-adjusted	Adjusted I	Adjusted II
I	ln HbA1c	Type of TB	9.26 (6.84, 12.52) < 0.0001	10.15 (7.30,14.10) < 0.0001	10.95 (7.51, 15.95) < 0.0001
II	ln CD3^+^T cell	Type of TB	0.56 (0.47, 0.67) < 0.0001	0.44 (0.36, 0.54) < 0.0001	0.39 (0.23, 0.68) 0.0008
III	ln CD3^+^ T cell	ln HbA1c categories	0.68 (0.55, 0.83) 0.0002	0.68 (0.54, 0.84) 0.0005	0.72 (0.52, 0.99) 0.0465

*Notes*:

Data are shown as OR (95% CI) and *p*.

Non-adjusted: no adjustment.

Adjusted I: adjusted for sex, age, and smoking.

Adjusted II of Model I: adjusted for sex, age, smoking, CD3^+^ T cells, CD19^+^ B cells, NK cells, AFS, neutrophils, ALB, and ADA.

Adjusted II of Model II: adjusted for sex, age, smoking, CD19^+^ B cells, NK cells, AFS, neutrophils, ALB, ADA, and HbA1c.

Adjusted II of Model III: adjusted for sex, age, smoking, CD19^+^ B cells, NK cells, AFS, neutrophils, ALB, and ADA.

Skewed CD3^+^ T cell and HbA1c data were log-transformed before analysis to meet normality assumptions.

The type of TB included PTB and PTB + EPTB.

ln HbA1c categories: ln(6%) = −2.813 was used as the cutoff to categorize the continuous ln HbA1c variable into controlled (≤ −2.813) and uncontrolled (> −2.813) groups, corresponding to the original 6% HbA1c threshold.

*Abbreviations*: TB, tuberculosis; HbA1C, glycated hemoglobin.

Model I showed a risk of HbA1c-driven dissemination. Each log unit increase in HbA1c (approximately 2.7% absolute) conferred a 10.95-fold higher risk of extrapulmonary dissemination (95% confidence interval [CI]: 7.51–15.95, *p* < 0.0001), which persisted after full adjustment (*n* = 1148). Demographic adjustment paradoxically amplified the estimate of risk (adjusted odds ratio [OR] = 10.15 – 10.95), which suggested negative confounding by age/sex/smoking.

Model II showed a protective effect of CD3^+^ T cells. Each log unit increase in CD3^+^ T cells (approximately 172% increase) reduced the dissemination risk by 61% (OR = 0.39, 95% CI: 0.23–0.68, *p* = 0.0008). The magnitude of protection increased with covariate adjustment (OR: 0.56–0.39), which indicated direct immunological effects and unmeasured pathways.

Model III showed metabolic–immune crosstalk. Higher CD3^+^ counts were associated with a 28% lower risk of elevated HbA1c levels (OR = 0.72, 95% CI: 0.52–0.99, *p* = 0.0465), which persisted with demographic (OR = 0.68) and full adjustments (*n* =1,148).

Models II and III showed bidirectional synergy between metabolic and immune factors. While HbA1c levels increased the dissemination risk (OR = 10.95), CD3^+^ T cells showed dual protective mechanisms through a reduction in direct dissemination (61%, Model II) and indirect glycemic mitigation (28%, Model III).

### 3.6 Threshold effect analysis of critical points in the associations between HbA1c and CD3^+^ T cells and TB dissemination

To test whether there are critical thresholds in the associations between HbA1c, CD3^+^ T cells, and TB dissemination, we used a segmented regression analysis using an iterative algorithm to identify potential inflection points. This method systematically evaluates different cutoff values to determine where the relationship between variables significantly changes in strength or direction. Our threshold effect analysis identified significant inflection points in three key relationships (Table 3).

**Table 3:** Threshold effect analysis of HbA1c and CD3^+^ T cells on the risk of TB dissemination.

Threshold model	Outcome	Exposure	Effect value (OR)	95% confidence interval (CI)	*p*
Model I	Type of TB	ln HbA1c	−2.01 (cutoff point)	−	−
		Below cutoff	1.79	(0.76, 4.23)	0.184
		Above cutoff	1078.01	(0.05, Inf)	0.169
Model II	Type of TB	ln CD3^+^T cell	5.96 (cutoff point)	−	−
		Below cutoff	11.26	(1.41, 89.58)	0.0222
		Above cutoff	0.23	(0.12, 0.44)	< 0.0001
Model III	ln HbA1c categories	ln CD3^+^T cell	7.65 (cutoff point)	−	−
		Below cutoff	0.67	(0.48, 0.94)	0.0190
		Above cutoff	7.87	(0.46, 135.21)	0.1553

*Notes*:

Model I was adjusted for sex, age, smoking, CD19^+^ B cells, NK cells, ALB, ADA, neutrophils, and ln CD3^+^ T cells.

Model II was adjusted for sex, age, smoking, CD19^+^ B cells, NK cells, ALB, ADA, neutrophils, and ln HbA1c.

Model III was adjusted for sex, age, smoking, CD19^+^ B cells, NK cells, ALB, ADA, and neutrophils.

Outcome of the type of TB: PTB and PTB + EPTB.

Outcome of ln HbA1c categories: ln(6%) = −2.813 was used as the cutoff to categorize the continuous ln HbA1c variable into the controlled (≤ −2.813) and uncontrolled (> −2.813) groups, corresponding to the original 6% HbA1c threshold.

Inf = infinity (the upper bound was not constrained in the estimation of extreme risk).

Bootstrap-derived 95% CIs were generated using 1,000 resamples.

ln HbA1c = −2.01 (corresponding to HbA1c = 7.38%), ln CD3^+^ T cells = 5.96 corresponds to CD3^+^ T cells = 387 cells/µL), and ln CD3^+^ T cells = 7.65 corresponds to CD3^+^ T cells = 2,100 cells/µL.

*Abbreviations:* TB, tuberculosis; OR, odds ratio; CI, confidence interval.

In Model I, the segmented regression showed a critical threshold at ln HbA1c = −2.01 (corresponding to HbA1c levels of approximately 7.4%). Below this threshold, HbA1c levels showed no significant association with TB dissemination (OR = 1.79, 95% CI: 0.76–4.23, *p* = 0.184). Above this threshold, each unit increase in ln HbA1c was associated with a substantial escalation in risk (OR = 1078.71, 95% CI: 0.05–infinity, *p* = 0.169), with the wide CI reflecting potential extreme risk of severe hyperglycemia.

Model II showed a biphasic pattern in the relationship between CD3^+^ T cells and TB dissemination, with a critical threshold at ln CD3^+^ T cells = 5.96 (approximately 387 cells/µL). Below this threshold, each log unit increase in CD3^+^ T cells was associated with an elevated risk of dissemination (OR = 11.26, 95% CI: 1.41–89.58, *p* = 0.022). However, above this threshold, elevated CD3^+^ T cells showed a protective effect, with a 77% reduction in the dissemination risk per log unit increase (OR = 0.23, 95% CI: 0.12–0.44, *p* < 0.0001).

In Model III, which examined the interaction between CD3^+^ T cells and HbA1c categories, the segmented regression identified a threshold at ln CD3^+^ T cells = 7.65 (corresponding to 2,100 cells/µL). Below this threshold, elevated CD3^+^ T cells showed a protective effect (OR = 0.67, 95% CI: 0.48–0.94, *p* = 0.019), while above this threshold, there was a non-significant trend toward an increased risk (OR = 7.87, 95% CI: 0.46–135.21, *p* = 0.155).

All models were appropriately adjusted for relevant demographic and clinical covariates (footnotes in Table 3).

## 4 Discussion

This study investigated the relationships between glycated hemoglobin, immune cell levels, and TB dissemination in a large cohort of 1,768 patients with TB. We used dual glycemic markers (HbA1c and FBG) along with detailed lymphocyte subset profiling. Initial analyses showed that poor glycemic control (HbA1c levels > 6% or FBG concentrations ≥ 7 mmol/L) was associated with a significant reduction in lymphocyte subsets in patients with PTB. A similar reduction was observed in patients with PTB + EPTB and HbA1c levels > 6%. Notably, patients with PTB + EPTB showed lower lymphocyte counts than those with PTB under controlled (HbA1c levels ≤ 6%) and uncontrolled glycemic conditions (HbA1c levels > 6%), particularly in T cell subsets. The multiple regression analysis further demonstrated that each log unit increase in HbA1c was associated with a 10.95-fold higher risk of extrapulmonary dissemination, while CD3^+^ T cells showed dual protective effects through a direct reduction in dissemination and indirect glycemic control. The threshold analysis identified three critical points: an HbA1c level of 7.4% for metabolic control, a CD3^+^ T cell count of 387 cells/µL (indicating immune deficiency), and a CD3^+^ T cell count of 2,100 cells/µL (indicating potential immune overactivation).

Our findings are consistent with previous studies that investigated the associations between HbA1c levels, a reduction in peripheral blood lymphocyte subsets, and TB. Antonio-Arques et al.^19^ and Lee et al.^20^ showed an increased risk of TB with poorer glycemic control, but neither study specifically addressed PTB + EPTB. Restrepo et al.^21^ and Kumar et al.^22^ found that chronic hyperglycemia was associated with altered cytokine profiles in patients with TB, but their small sample sizes and lack of T/B cell subset analysis limited their conclusions. Li et al.^23^ reported reduced CD4^+^ and CD8^+^ T cell counts in patients with TB, which is consistent with our findings, but our study expands on their study by including HbA1c, FBG concentrations, and B cell analysis in a larger cohort. Our threshold analysis identified three critical points with important clinical implications. The HbA1c threshold of 7.4% is in agreement with the American College of Physicians recommended glycemic control levels of 7%–8%^24^, supporting the importance of glycemic control in managing TB. The low CD3^+^ T cell threshold (387 cells/µL) found in our study corresponds with lymphopenia patterns observed in severe viral infections, in which 34%–41% of patients with severe influenza and severe acute respiratory syndrome coronavirus 2 show CD3^+^ T cell counts < 400 cells/µL.^25^ This finding suggests a common immunocompromised state predictive of pathogen dissemination. In our study, the upper CD3^+^ T cell threshold (2,100 cells/µL) and its paradoxical effect are consistent with previous findings of immune-mediated insulin resistance,^26^ highlighting the complex immune–metabolic interactions in the comorbidity of TB and diabetes.

Mechanistically, hyperglycemia in patients with DM suppresses immune function through various mechanisms, such as the accumulation of advanced glycation end products, increased oxidative stress, and excessive expression of inflammatory cytokines.^27^ These mechanisms likely contribute to a considerable reduction in T cells, B cells, and NK cell function, thereby increasing the risk of TB dissemination.

This study has some strength as follows. These strengths include the large retrospective cohort (*n* = 1,768) and comprehensive analysis using dual glycemic markers (HbA1c and FBG) along with detailed lymphocyte profiling to investigate the mechanisms of TB dissemination. The robust statistical approach, incorporating multiple regression analyses with extensive confounder adjustment, strengthens the reliability of our findings. Most notably, our threshold effect analysis identified clinically actionable cutoff points, which comprised an HbA1c level of 7.4% for metabolic control, CD3^+^ T cell count of 387 cells/µL, indicating immune deficiency, and CD3^+^ T cell count of 2,100 cells/µL, indicating potential immune overactivation. The identification of bidirectional metabolic-immune interactions (61% direct protection and 28% indirect glycemia-associated protection from CD3^+^ T cells) offers novel insights for developing integrated treatment strategies. These thresholds provide guidance for clinical practice, which could enable risk stratification and targeted interventions in managing comorbidity of TB and diabetes. On the basis of our findings, we recommend the following clinical strategies Patients with diabetes and an HbA1c value ≥ 7.4% should be screened for EPTB because this threshold indicates a 10.95-fold increased risk. Additionally, CD3^+^ T cell counts should be monitored in immunocompromised patients, and intervention should be performed when the cell count drops below 387 cells/µL, which is associated with an 11.26-fold elevation in risk. Glycemic control should be optimized to maintain HbA1c values < 7.4% and preserve the CD3^+^ T cell count at > 387 cells/µL in populations prone to TB. Furthermore, physicians should be vigilant for elevated HbA1c levels when CD3^+^ T cells exceed 2,100 cells/µL because this may indicate immune-mediated insulin resistance.

Limitations to this study include the single-center design, which may limit generalizability, and the predominantly Chinese cohort, necessitating validation in other populations. Additionally, the observational design of this study precludes causal inferences, warranting cautious interpretation. Future research needs to focus on conducting multinational studies to enhance generalizability, implementing prospective interventional studies to establish causal relationships, and investigating molecular mechanisms using advanced techniques such as single-cell sequencing.

## 5 Conclusion

Poor glycemic control is associated with considerable depletion of CD3^+^, CD4^+^, and CD8^+^ T cells and CD19^+^ B cells in patients with PTB and in those with PTB + EPTB. Extrapulmonary dissemination further exacerbates immune dysfunction, with a reduction in T cell subsets, regardless of the glycemic status. Each log unit increase in HbA1c is associated with a 10.95-fold higher risk of extrapulmonary dissemination, while CD3^+^ T cells show dual protective effects through a direct reduction in dissemination and indirect improvement in glycemic control. Three clinically actionable cutoff points were identified in this study: an HbA1c value of 7.4% for metabolic control, a CD3^+^ T cell count of 387 cells/µL, indicating immune deficiency, and a CD3^+^ T cell count of 2,100 cells/µL, indicating potential immune overactivation. These findings provide guidance for risk stratification and targeted interventions in managing comorbidity of TB and diabetes.

## CRediT authorship contribution statement

**Yujun Lin:** Writing – original draft, Software, Investigation, Formal analysis, Data curation, Funding acquisition, Resources, Visualization. **Xiaohong Chen:** Supervision, Funding acquisition, Validation. **Jiangwei Chen:** Data curation. **Di Wu:** Supervision, Conceptualization, Funding acquisition, Methodology, Project administration, Writing – review & editing.

## Appendix Supplementary materials

Image, application 1
