# Supplementary material for: Effect of glycemic control on lymphocyte subsets in the dissemination of pulmonary tuberculosis: A retrospective analysis
Source: Infect Med (Beijing). 2025 May 17;4(2):100183. doi: 10.1016/j.imj.2025.100183 (PMC12173055; doi:10.1016/j.imj.2025.100183)
Supplement: Supplementary file 1 [file mmc1.docx]

***Supplementary result***

***1. PTB+EPTB Subgroup Analysis Under Different HbA1c Levels***

In PTB + TBP subgroup, the HbA1c > 6% group exhibited lower counts of CD3^+^ T cells (747 vs. 786, *p* = 0.3299), CD4^+^ T cells (446.5 vs. 469, *p* = 0.4912), CD8^+^ T cells (268 vs. 303, *p* = 0.1743), CD19^+^ B cells (141 vs. 144, *p* = 0.2421), and CD16^+^CD56^+^ NK cells (160 vs. 166, *p* = 0.6073) compared to the HbA1c ≤ 6% group, but the differences were not statistically significant (Fig. S1A–E).

In PTB + BTB subgroup, the HbA1c > 6% group had significantly lower counts of CD8^+^ T cells (322.5 vs. 403, *p* = 0.0282) and CD19^+^ B cells (18.5 vs. 294, *p* < 0.0001) compared to the HbA1c ≤ 6% group (Fig. S1H–I). However, the differences in CD3^+^ T cells (1116 vs. 1136, *p* = 0.4167), CD4^+^ T cells (705.5 vs. 650, *p* = 0.6997) and CD16^+^CD56^+^ NK cells (186 vs. 173, *p* = 0.7989) were not statistically significant between the two groups (Fig. S1F, G, J).

In PTB + M-EPTB subgroup, the HbA1c > 6% group exhibited significantly lower counts of CD3^+^ T cells (879.5 vs. 1082, *p* = 0.0246) and CD4^+^ T cells (446.5 vs. 640, *p* = 0.0040) compared to the HbA1c ≤ 6% group (Fig. S1K–L). Interestingly, the count of CD16^+^CD56^+^ NK cells was higher in the HbA1c > 6% group (237 vs.135, *p* = 0.0052) (Fig. S1O), while the differences in CD8^+^ T cells (283 vs. 398.5, *p* = 0.3712) and CD19^+^ B cells (174 vs. 199, *p* = 0.1084) were not statistically significant (Fig. S1M–N).


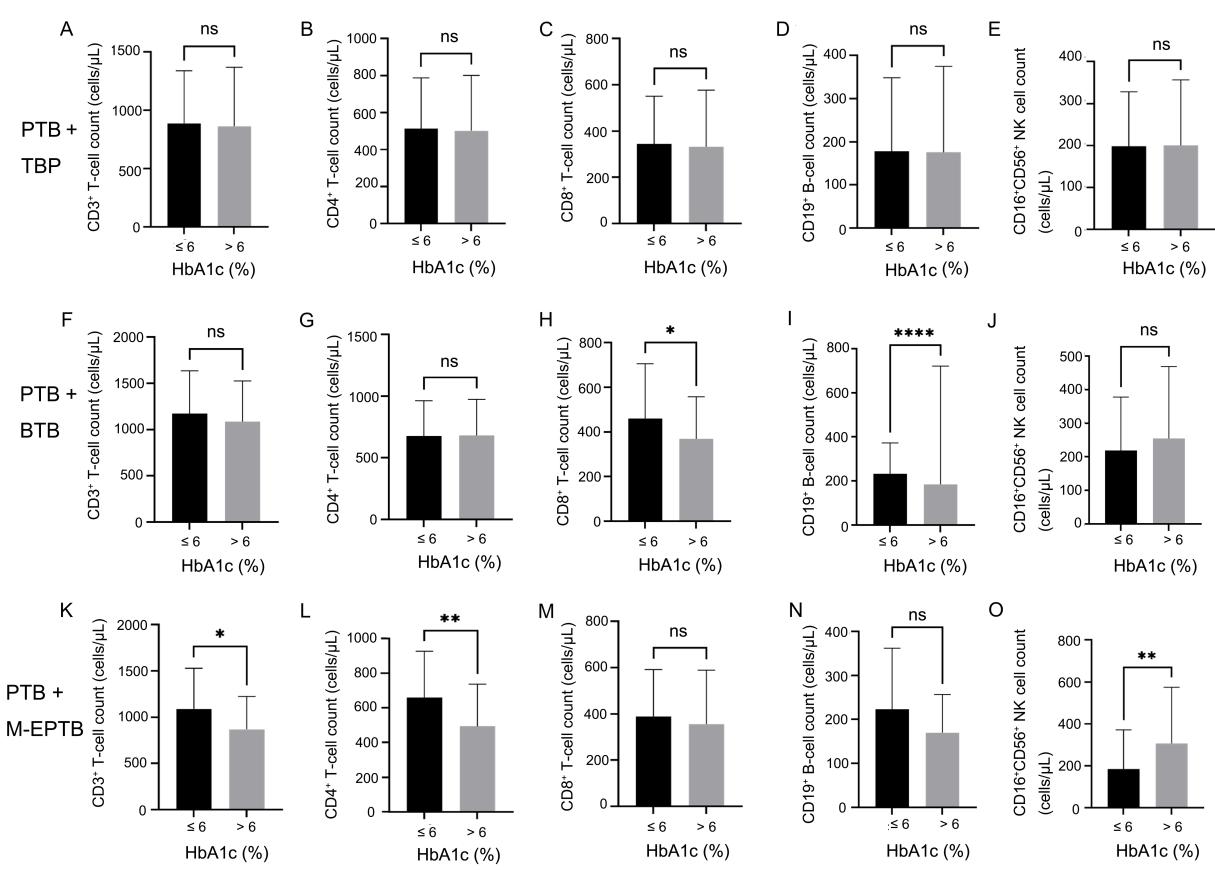


Fig.S1. Comparison of TBNK subset cell counts at the time of admission across different HbA1c levels in sugroups of EPTB. **p* < 0.05; ***p* < 0.01; *****p* < 0.0001; ns, not significant.

*Abbreviations*: HbA1c, glycated hemoglobin; PTB, pulmonary tuberculosis; TBP, tuberculous pleritis; BTB, bronchial tuberculosis; M-EPTB, miscellaneous EPTB; TBNK, T cells, B cells, and natural killer cells.

***2. PTB group analysis under different FBG levels***

***
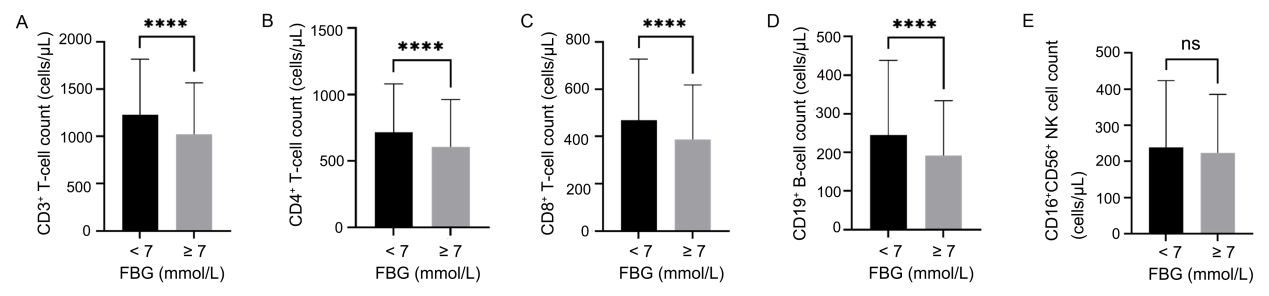
***

**Fig. S2.** Comparison of TBNK subset cell counts at the time of admission across different FBG levels in PTB gourp. *****p* < 0.0001; ns, not significant.

*Abbreviations*: FBG, fasting blood glucose; PTB, pulmonary tuberculosis; TBNK, t cells, b cells, and natural killer cells.

***3. Comparison of TBNK Cell Subsets between the PTB and PTB+EPTB Groups with FBG Concentrations < 7 mmol/L***

***
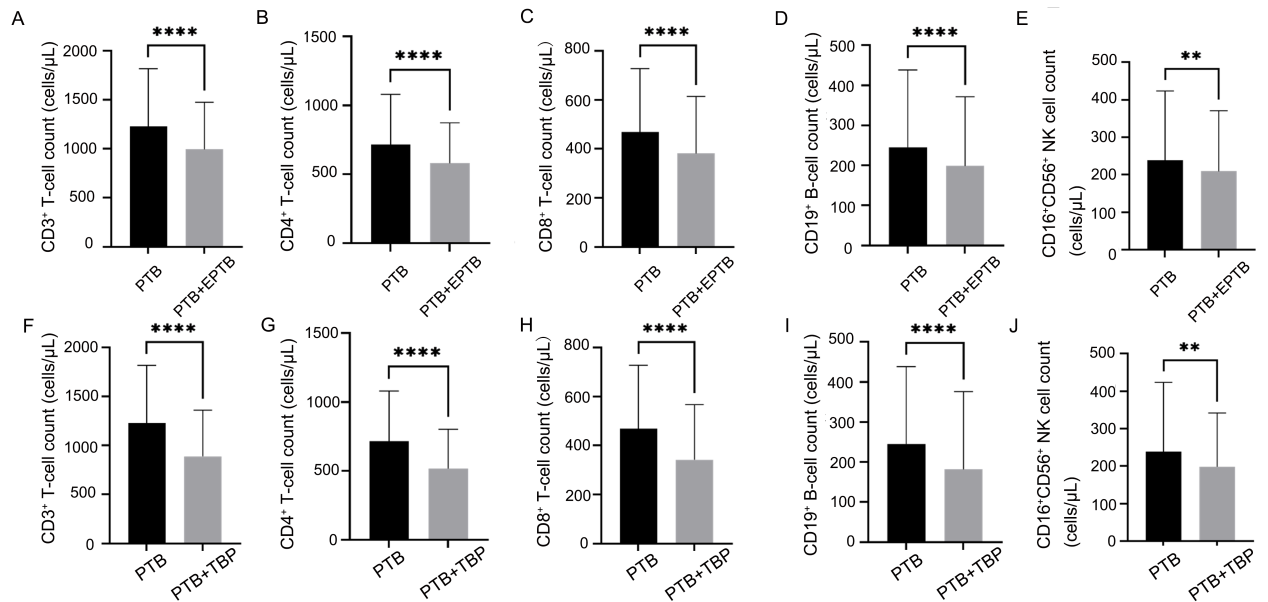
***

**Fig. S3.** Comparison of TBNK subset cell counts between PTB and PTB+EPTB as well as PTB and PTB+TBP when FBG ＜ 7 mmol/L. ***p* < 0.01; *****p* < 0.0001.

*Abbreviations*: PTB, pulmonary tuberculosis; EPTB, extrapulmonary tuberculosis; TBP, tuberculosis pleuritis; TBNK, T cells, B cells, and natural killer cells.

***4. Comparison of TBNK cell subsets between PTB and other EPTB subgroups (PTB vs. PTB + BTB and PTB vs. PTB + M-EPTB) under good glycemic control (HbA1c ≤ 6% and FBG ＜ 7 mmol/L )***

When comparing the PTB group to the PTB+BTB subgroup in patients with HbA1c ≤ 6%, the PTB+BTB group exhibited lower levels of CD3^+^ T cells, CD4^+^ T cells, CD8^+^ T cells, CD19^+^ B cells and CD16^+^CD56^+^ NK cells, but the differences were not statistically significant (1,136 vs. 1,166, *p* = 0.352; 650 vs. 683, *p* = 0.2694; 403 vs. 440, *p* = 0.5190; 204 vs. 211, *p* = 0.9043; 173 vs. 182, *p* = 0.4176, respectively; Fig. S4A–E).

Similarly, in the comparison between PTB and the PTB+M-EPTB subgroup with HbA1c ≤ 6%, the PTB+M-EPTB group showed significantly lower levels of CD8^+^ T cells (398.5 vs. 440, *p* = 0.0414) and CD16^+^CD56^+^ NK cells (135 vs.182, *p* = 0.0072), while the reductions in CD3^+^ T cells, CD4^+^ T cells and CD19^+^ B cells were not statistically significant (1,082 vs. 1,166, *p* = 0.1096; 640 vs. 683, *p* = 0.5200; 199 vs. 211, *p* = 0.5454, respectively; Fig. S4F–J).


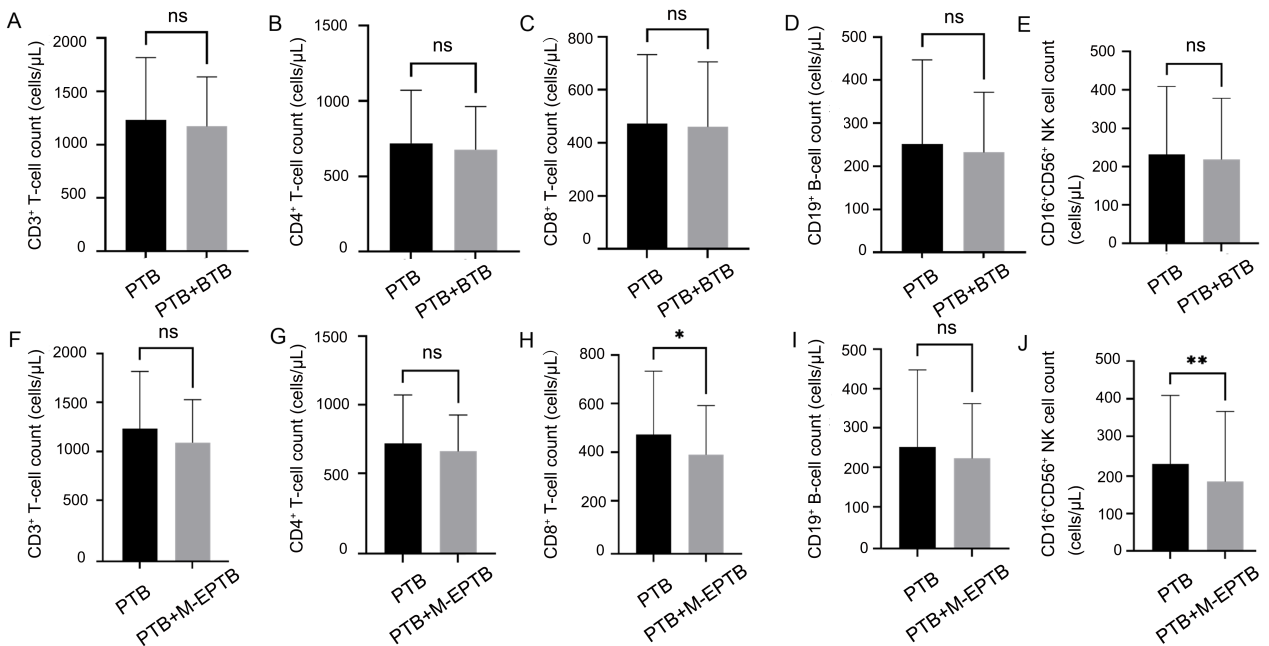


**Fig.S4.** Comparison of TBNK subset cell counts between PTB and PTB+BTB as well as PTB and PTB+M-EPTB when HbA1c ≤ 6%. **p* < 0.05; ***p* < 0.01; ns, not statistically significant.

*Abbreviations*: PTB, pulmonary tuberculosis; BTB, bronchial tuberculosis; M-EPTB, miscellaneous EPTB; TBNK, t cells, b cells, and natural killer cells.

By incorporating FBG measurements as an additional metabolic parameter, we found that when comparing the PTB group to the PTB+BTB subgroup in patients with FBG ＜ 7 mmol/L, the PTB+BTB group exhibited lower levels of CD3^+^ T cells, CD4^+^ T cells, CD8^+^ T cells, CD19^+^ B cells and CD16^+^CD56^+^ NK cells, but the differences were not statistically significant (1,139 vs. 1,147, *p* = 0.4023; 654.5 vs. 674.5, *p* = 0.4444; 403 vs. 432.5, *p* = 0.4688; 198.5 vs. 207, *p* = 0.8734; 174 vs. 184, *p* = 0.3307, respectively).

Similarly, in the comparison between PTB and the PTB+M-EPTB subgroup with FBG ＜ 7 mmol/L, the PTB+M-EPTB group showed significantly lower levels of CD3^+^ T cells (999 vs. 1,147, *p* = 0.0349) and CD8^+^ T cells (373.5 vs. 432.5, *p* = 0.0151), while the reductions in CD4^+^ T cells, CD19^+^ B cells and CD16^+^CD56^+^ NK cells were not statistically significant (614 vs. 674.5, *p* = 0.1795; 188 vs. 207, *p* = 0.2636; 148.5 vs. 184, *p* = 0.0968, respectively).

***5. Comparison of TBNK cell subsets in PTB vs. PTB + TBP groups among subjects with FBG levels ≥ 7 mmol/L.***


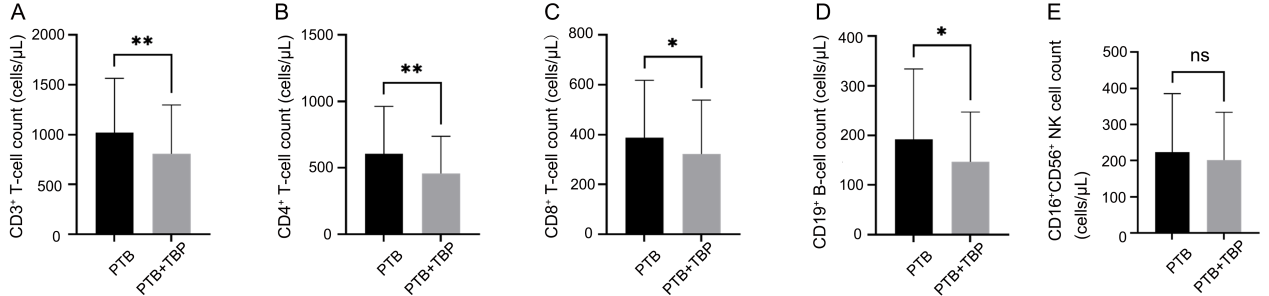


**Fig. S5.** Comparison of TBNK subset cell counts between PTB and PTB+TBP when FBG ≥ 7 mmol/L. **p* < 0.05; ***p* < 0.01; ns, not significant.

*Abbreviations*: PTB, pulmonary tuberculosis; EPTB, extrapulmonary tuberculosis; TBP, tuberculosis pleuritis; TBNK, t cells, b cells, and natural killer cells.

***6. Comparison of TBNK cell subsets between PTB and other subgroups of EPTB (PTB vs. PTB + BTB and PTB vs. PTB + M-EPTB) under poor glycemic control (HbA1c > 6% and FBG ≥ 7 mmol/L)***

When HbA1c was > 6%, the PTB+BTB subgroup showed no statistically significant differences in TBNK subsets compared to the PTB group (CD3^+^ T cells: 1,116 vs. 964, *p* = 0.4307; CD4^+^ T cells: 705 vs. 503, *p* = 0.1493; CD8^+^ T cells: 322.5 vs. 356, *p* = 0.4801; CD19^+^ B cells: 160 vs. 166, *p* = 0.6868; CD16^+^CD56^+^ NK cells: 186 vs. 178, *p* = 0.8588; Fig. S6 A–E).

Similarly, when HbA1c was > 6%, the PTB + M-EPTB subgroup did not exhibit statistically significant differences in TBNK subsets compared to the PTB group (CD3^+^ T cells: 879.5 vs. 964, *p* = 0.1082; CD4^+^ T cells: 446.5 vs. 573, *p* = 0.0603; CD8^+^ T cells: 283 vs. 356, *p* = 0.1868; CD19^+^ B cells: 174 vs. 166, *p* = 0.8754; CD16^+^CD56^+^ NK cells: 237 vs.178, *p* = 0.1529; Fig. S6 F–J).


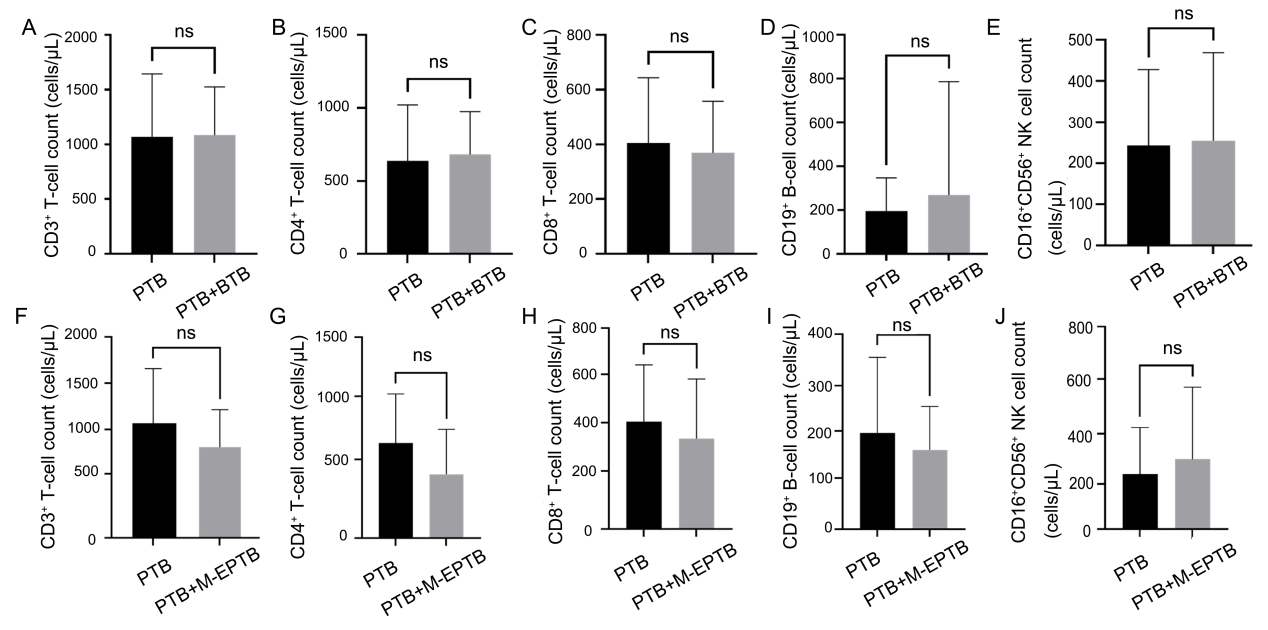


**Fig. S6.** Comparison of TBNK subset cell counts between PTB and PTB + BTB as well as PTB and PTB + M-EPTB when HbA1c ＞ 6%. ns, not significant.

*Abbreviations*: PTB, pulmonary tuberculosis; BTB, bronchial tuberculosis; M-EPTB, miscellaneous EPTB; TBNK, T cells, B cells, and natural killer cells.

As FBG was utilized as a complementary glycemic control marker. We found that when FBG ≥ 7 mmol/L, the PTB + BTB subgroup showed no statistically significant differences in TBNK subsets compared to the PTB group (CD3^+^ T cells: 1,101 vs. 939.5, *p* = 0.1753; CD4^+^ T cells: 645.5 vs. 541, *p* = 0.1136; CD8^+^ T cells: 343 vs. 340.5, *p* = 0.8728; CD19^+^ B cells: 182.5 vs. 165, *p* = 0.1573; CD16^+^CD56^+^ NK cells: 178 vs. 176, *p* = 0.9995).

Similarly, the PTB + M-EPTB subgroup did not exhibit statistically significant differences in TBNK subsets compared to the PTB group (CD3^+^ T cells: 949 vs. 939.5, *p* = 0.5328; CD4^+^ T cells: 461 vs. 541, *p* = 0.2358; CD8^+^ T cells: 301 vs. 340.5, *p* = 0.6186; CD19^+^ B cells: 177 vs. 165, *p* = 0.5660; CD16^+^CD56^+^ NK cells: 187 vs.176, *p* = 0.7684).

**Table S1 Multiple regression analysis**

| Exposure | Non-adjusted | Adjusted |
| --- | --- | --- |
| CD4^+^ T cells | 0.60 (0.51, 0.71) < 0.0001 | 0.68 (0.53, 0.88) 0.0030 |

*Notes*:

Data are shown as OR (95% CI) and *p*.
Outcome Variable: Type of TB (0 = PTB, 1 = PTB+EPTB)
Exposure Variable: CD4^+^ T cells
Non-adjusted Model Adjusted for: None
Adjusted Model Adjusted for: CD8^+^ T cells, CD19^+^ B cells and CD16^+^CD56^+^ NK cells

Sample Sizes for Each Model

| Outcome | Exposure | Non-adjusted | Adjusted |
| --- | --- | --- | --- |
| Type of TB | CD4^+^ T cells | 1690 | 1688 |

*Note*: This table was generated using EasyStat software ([www.empowerstats.com](http://www.empowerstats.com/)) and R software.

**Table S2 Multiple regression analysis**

| Exposure | Non-adjusted | Adjusted |
| --- | --- | --- |
| CD8^+^ T cells | 0.62 (0.53, 0.73) < 0.0001 | 0.75 (0.61, 0.93) 0.0077 |

*Notes*:

Data are shown as OR (95% CI) and *p*.
Outcome Variable: Type of TB (0 = PTB, 1 = PTB+EPTB)
Exposure Variable: CD8^+^ T cells
Non-adjusted Model Adjusted for: None
Adjusted Model Adjusted for: CD4^+^ T cells, CD19^+^ B cells and CD16^+^CD56^+^ NK cells

Sample Sizes for Each Model

| Outcome | Exposure | Non-adjusted | Adjusted |
| --- | --- | --- | --- |
| Type of TB | CD8^+^ T cells | 1690 | 1688 |

This table was generated using EasyStat software ([www.empowerstats.com](http://www.empowerstats.com/)) and R software.

**Table S3 Multiple regression analysis**

| Exposure | Non-adjusted | Adjusted I | Adjusted II |
| --- | --- | --- | --- |
| CD3^+^ T cells | 0.80 (0.57, 1.10) 0.1719 | 0.52 (0.36, 0.75) 0.0005 | 0.39 (0.22, 0.67) 0.0008 |

*Notes*:

Data are shown as OR (95% CI) and *p*.
Outcome Variable: Type of TB (0 = PTB, 1 = PTB+EPTB)
Exposure Variable: CD3^+^ T cells
Non-adjusted Model Adjusted for: None
Adjusted I Model adjusted for: Sex; Age; Smoking

Adjust II model adjusted for: SEX; AGE; Smoking; CD19^+^ B cells; NK cells; AFS; Nutrofil; ALB; ADA

Use subset of data: ln HbAc > −2.813（HbA1c ＞ 6%）

This table was generated using EasyStat software ([www.empowerstats.com](http://www.empowerstats.com/)) and R software.
